# A Low Temperature Coefficient Time-to-Digital Converter with 1.3 ps Resolution Implemented in a 28 nm FPGA

**DOI:** 10.3390/s22062306

**Published:** 2022-03-16

**Authors:** Xiangyu Mao, Fei Yang, Fang Wei, Jiawen Shi, Jian Cai, Haiwen Cai

**Affiliations:** 1 Key Laboratory of Space Laser Communication and Detection Technology, Shanghai Institute of Optics and Fine Mechanics, Chinese Academy of Sciences, Shanghai 201800, China; xymao@siom.ac.cn (X.M.); weifang@siom.ac.cn (F.W.); shijiawen@siom.ac.cn (J.S.); hwcai@siom.ac.cn (H.C.); 2 Institute of Microelectronics, Chinese Academy of Sciences, Beijing 100029, China; caijian@ime.ac.cn

**Keywords:** field programmable gate array (FPGA), time to digital converter (TDC), multichain segmentation, low temperature coefficient

## Abstract

Time-to-digital converter (TDC) is the key technology to realize accurate time delay measurement in high-precision optical fiber time-frequency transmission and synchronization, optical sensing and many scientific applications. The performance of FPGA-TDC based on the carry chain is sensitive to the operating temperature. This paper presents a parallel multichain cross segmentation method, without multitime measurements, which merges multichain into an equivalent chain, achieving low temperature coefficient and maintaining high precision. The equivalent chain breaks the limit of the intrinsic cell delay of a single carry chain, improves the precision and reduces the impact of temperature variation significantly. A two-channel TDC based on parallel multichain cross segmentation method is implemented in a 28 nm fabrication process Kintex-7 FPGA. The results show that the performance of TDC is improved with the increase of the number of chains. The 10-chain TDC with 1.3 ps resolution, 4.6 ps single-shot precision performs much better than the plain TDC with 11.4 ps resolution, 8.7 ps single-shot precision. The resolution is stable with 0.0002 ps/°C temperature coefficient under an operating temperature range from 25 °C to 70 °C. Moreover, the proposed method reduces the complexity of the circuit and the resource usage.

## 1. Introduction

Accurate time interval measurement is applied in high precision time synchronization, optical sensing and many scientific applications. High-precision optical fiber time-frequency transmission and synchronization technology is the most accurate time-frequency synchronization technology currently in widescale use in the fields of clock comparison [1,2], radar networking [3], and basic scientific research [4]. The time-of-flight (TOF) is measured in Light Detection and Ranging(LiDAR) [5], optical sensing systems for depth measurement [6] and so on. The electric pulse from the optical sensing system is converted into the optical signal which travels toward the illuminated object and is reflected back to the optical sensing system. The measurement precision of the round-trip delay time of the optical signal affects the performance of the system. Commercial instruments are commonly used for time interval measurement, such as SR620 time interval analyzer of the SRS company, with a measurement precision of 25 ps [7]. In order to meet more and more ps-level performance and integrated requirements, a time-to-digital converter (TDC) is implemented for application in specific integrated circuits or in field programmable gate arrays. Acam’s TDC-GPX2 dedicated chip can achieve 10 ps resolution in M mode [8], while the latest prototype chip of CERN Microelectronics Group can achieve 3 ps precision [9]. In recent years, the FPGA-TDC has been widely studied and used due to the advantages of flexibility, short development time and low cost. There are three commonly used approaches to implement high-performance FPGA-TDC: (1) multiphase method, which uses phase differences between multiple clocks as the interpolation mechanism to achieve high resolution [10,11]; (2) Vernier method, in which the resolution is given by the difference between two delay lines [12]; and (3) the tapped delay line (TDL) method, which uses FPGA’s cells as the interpolator to improve the resolution. In the past few years, TDL has become the main implementation method of FPGA-TDC [13,14,15,16,17,18,19,20,21,22,23,24,25,26,27,28,29]. In 2006, Song et al. used dedicated carry chain as TDL for time interpolation, achieving a TDC with 69.5 ps resolution and 65.8 ps precision in the Virtex-II FPGA [18]. In 2008, Wu et al. proposed a novel wave union architecture to achieve a TDC with 10 ps precision in the Cyclone II FPGA through 16 measurements [19,20]. In 2011, Bayer et al. designed a multichain parallel architecture TDC, which improves the resolution by averaging the measurements from multiple parallel chains [21]. In 2017, Qin et al. obtained a 1.15 ps resolution and 3.5 ps precision TDC with 20 chains in the Virtex-7 FPGA based on multichain measurements averaging method [22].

There are two main factors limiting the performance of FPGA-TDC based on carry chain. The bins of carry chain are non-uniformity because of manufacturing process technology and have some ultrawide values due to extra route and clock skew. In order to get high-performance of TDC, some architectures are proposed. The wave union method utilizes multiple measurements in a single chain to reach time resolution beyond the intrinsic carry cell delay, which increases dead time to complete multiple measurements and has a complex encoder [23]. Some applications need high sampling rates and the increasing number of resources available in FPGA has contributed to the research of multichain architecture [30], architecture which uses multiple independent TDLs to measure the input signal, with each chain generating a timestamp. The performance can be improved by averaging all the timestamps.

The delay time of a carry chain is sensitive to the manufacturing process and power supply voltage and operating temperature (PVT), thus the measurement precision and accuracy could deteriorate due to voltage and temperature variation [22,24]. A high stability power supply can be used to reduce voltage fluctuation, but the temperature variation is commonly inevitable. Therefore, a special temperature correction module/strategy is needed. One method is to obtain the fitting relationship between temperature and bin size at an early stage with extensive measurements [22], then the temperature coefficient is compensated to realize the temperature correction. Another method is to control TDC to update a look-up table (LUT) containing the actual bin size, through bin-by-bin calibration, as the temperature changes [24,25,26]. However, real-time and accurate temperature corrections are difficult to implement at the same time and the extra component will increase the complexity and resource consumption of the circuit.

This paper presents a parallel multichain cross segmentation method without multitime measurements. The TDC implemented by this method has the following advantages:

Firstly, the TDC has a very high performance. The parallel multichain cross segmentation method merges multichain into an equivalent chain through mutual segmentation between chains, which divide the ultrawide bin into different smaller bins. Therefore, the equivalent chain breaks the limit of the intrinsic cell delay of the plain chain. The performance of TDC is improved as the number of chains increases. Finally, the 10-chain TDC obtains a 1.3 ps resolution and a 4.6 ps single-shot precision. The dead time of the TDC is two system clock cycles.

Secondly, the TDC has a low temperature coefficient. The drift of the delay time of the cell in the plain chain due to the temperature fluctuation is divided by other delay chains with the same drift, which weakens the impact of the temperature fluctuation on the plain chain and makes the equivalent chain insensitive to the temperature fluctuation. Therefore, TDC has a low temperature coefficient without dedicated temperature correction component.

Finally, the method proposed in this paper makes circuit compactness and stability and requires low resource usage. The architecture makes TDC insensitive to temperature without any additional correction logic. In essence, both wave union and multichain methods average the results after multiple measurements of the same event to improve the precision. In the multichain method, each chain needs an LUT to store the actual bin size obtained by bin-by-bin calibration. It also needs to test and store the delay time between different chains. In the proposed method, we can get the timestamp directly from the equivalent chain without multitime measurements.

## 2. Methodology

**Figure 1:**
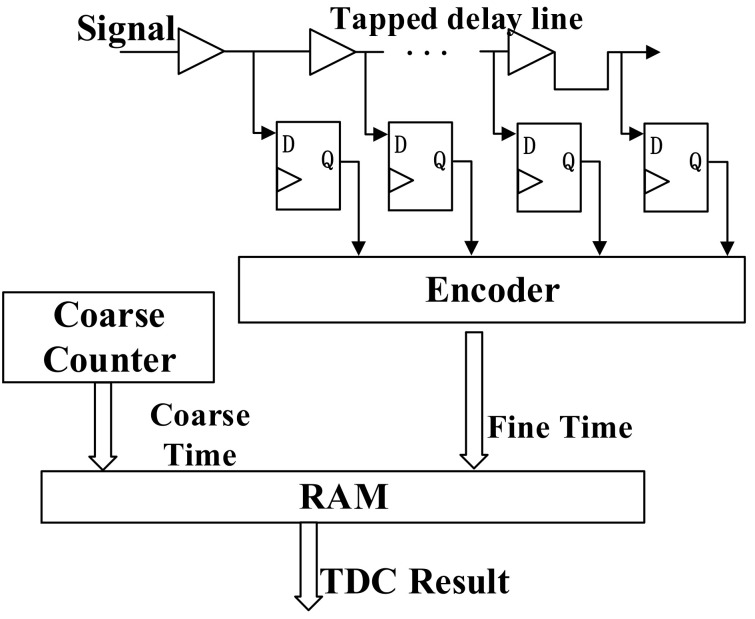
Block diagram of TDL-based TDC.

The architecture of the FPGA-TDC implemented with TDL interpolation is shown in Figure 1. The coarse counter is used to record the number of the clock period, achieving a wide range time interval measurement. TDL is used for interpolation, measuring less than one clock period as fine time. The resolution of the TDC is the cell width of TDL. When a 0-to-1 transition occurs to the input signal, the D-flip-flop group begins to convert 0–1. At the next rising edge of the clock, the D-flip-flop group obtains a thermometer code. The encoder converts the number of 1 in the thermometer code into a binary number that represents how many delay units are inserted in the time interval between the input signal arrival and the next rising edge of the clock.

**Figure 2:**
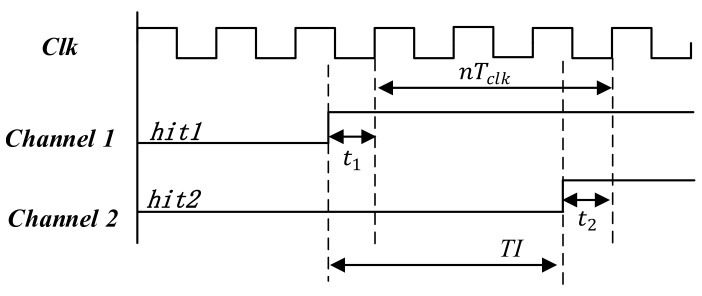
Timing diagram of time interval measurement.$\;nT_{clk}$ is the coarse time. $t_{1}$ and $t_{2}$ are the fine times, and *TI* is the time interval between *hit1* and *hit2*.

The TDC produces a timestamp when a hit signal is fed into a channel. The timing diagram of two hit signals is shown in Figure 2. The time interval between hit1 and hit2 is calculated from coarse and fine times as follows:

(1) $$TI=nT_{clk}+t_{1}-t_{2}.$$

where $nT_{clk}$ means the integer number n of the clock period that appears between hit1 and hit2 and which are fed into two channels. $\;t_{1}$ and $t_{2}$ represent the time interval between hit1, hit2 and the nearest edge of the clock, respectively.

### 2.1. Bin-by-Bin Calibration

**Figure 3:**
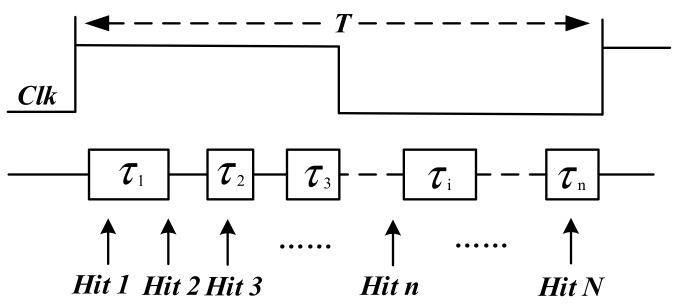
Model diagram of bin-by-bin calibration for carry chain. $\tau_{i}$ represents the size of the *i*-th bin. *Hit n* is the random calibration signal distributed within the range of one clock period.

The TDL construct of the carry chain inside FPGA is non-uniformity because of manufacturing process technology and has some ultrawide bins due to extra route and clock skew, which results in large nonlinearity and poorer precision. Therefore, calibration for carry chain is required to obtain accurate measurements. There is a statistical approach that can provide bin-by-bin calibration [17]. As shown in Figure 3, $\tau_{i}$ represents the size of the *i*-th bin. Hits are random signals distributed within the range of the clock period (0, *T*), so the time interval between the hit and the rising edge of the clock is completely random. The number of hits captured by the bin is proportional to its size. When a large number of hits are fed into the chain, we can use the counts $n_{i}$ captured by the *i*-th bin as its size. Assuming the total number of hits is *N*, the size of the *i*-th bin is:

(2) $$\tau_{i}=\frac{n_{i}}{N}T.$$

**Figure 4:**
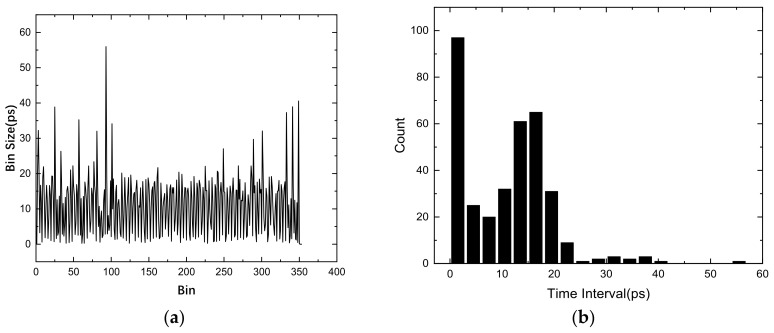
Bin size of plain TDC (**a**) bin sizes tested using bin-by-bin calibration (**b**) histogram of the bin size.

The bin size of carry chain in the Xilinx Kintex-7 device is show in Figure 4a. According to Figure 4b, the typical bin size is approximately 15 ps, while the largest bin size is 56 ps, and approximately 100 bins have a small size because of the non-uniformity in the carry chain and because of differences in clock skew on the clock distribution tree.

The time interval between hit and rising edge of the clock is

(3) $$t_{j}=\sum_{i=0}^{j}\tau_{i}.$$

The measurement error is one bin size, so the true value $t^{\prime }\in \left(t_{j-1},t_{j-1}+\tau_{i}\right)$.The standard deviation of the measurement error is

(4) $$\sigma^{2}=\frac{1}{t_{j}-t_{j-1}}\int_{t_{j-1}}^{t_{j}}{\left(t-t^{\prime }\right)}^{2}dt=\frac{{\left(t_{j}-t^{\prime }\right)}^{3}-{\left(t_{j-1}-t^{\prime }\right)}^{3}}{3\left(t_{j}-t_{j-1}\right)}.$$

where $t^{\prime }=t_{j-1}+(t_{j}-t_{j-1})/2$,$\;d\sigma^{2}/dt^{\prime }=0$. In order to get the minimum error, we define $t_{j}$ as

(5) $$t_{j}=\sum_{i=0}^{j-1}\tau_{i}+\tau_{j}/2.$$

### 2.2. Effects of Temperature and Thermally Induced Electronic Noise

The logic cell of FPGA is based on a static random-access memory (SRAM) process composed of transistors. We can use a complementary metal oxide semiconductor (CMOS) inverter as a model to analyze the effect of temperature on the chain delay variation inside the FPGA chip [25]. The transmission delay of inverter in CMOS can be approximately expressed as

(6) $$t_{p}=0.69R_{eq}\left(T\right)C_{L}\left(T\right).$$

where $R_{eq}\left(T\right)$ is the average “on” resistance of the transistor and $C_{L}\left(T\right)$ is the load capacitance.

Although carry chain is more complex than the CMOS inverter model, the basic principle is the same. In the FPGA chip, due to the increase of the routing to the carry cells at the boundary, the $C_{L}\left(T\right)$ increases, resulting in large transmission delay. Theoretically, carrier mobility and threshold voltage which are related to temperature, affect the $R_{eq}\left(T\right)$, and $C_{L}\left(T\right)$ is also affected by temperature. Thus, transmission delay $t_{p}$ varies with temperature. The bin size of a carry chain is closely related to the transmission delay. Therefore, the bin size of a carry chain is also affected by temperature.

During the working process, the temperature of the chip changes due to the working heat or the change of the external heat source, thereby introducing thermally induced electronic noise, which is reflected in the change of the bin size. The drift of the bin size will bring extra quantization errors, which will deteriorate the accuracy of the measurement. In previous work, J. Wang et al. [24] indicated that the temperature coefficient of averaged bin size of a Xilinx virtex-4 device is 0.047 ps/°C with the temperature range from 31 °C to 61 °C. In our later experiments, the temperature coefficient of averaged bin size of Xilinx kintex-7 device is 0.014 ps/°C with the temperature range from 25 °C to 70 °C.

### 2.3. Multichain Cross Segmentation Method

The multichain cross segmentation method will be descripted from three aspects as follows.

**Figure 5:**
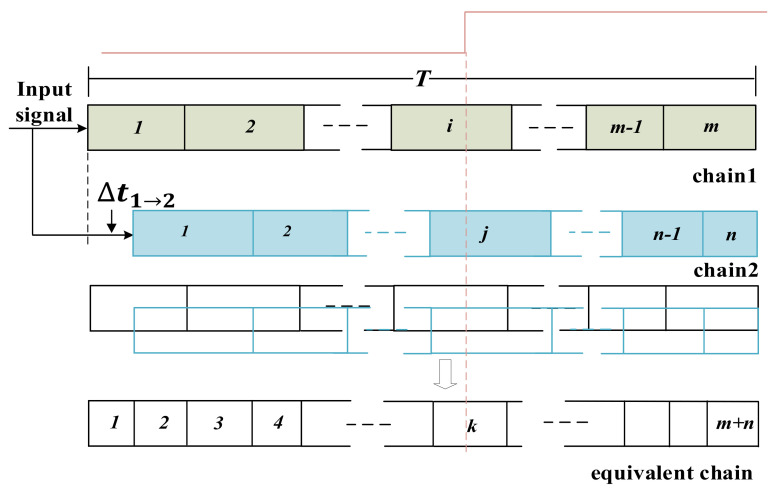
Schematic diagram of cross segmentation with two chains.

***The principle and implementation of multichain cross segmentation method.*** As mentioned above, non-uniform and ultrawide bins of the carry chain limit the precision of TDC, and temperature fluctuation deteriorates measurement accuracy. We subdivide the ultrawide bin by mutual segmentation between parallel chains, and finally merge all the chains into an equivalent chain. The bin size of the equivalent chain is smaller and more uniform, which breaks the limit of the intrinsic cell delay of single chain and is temperature tolerant. It is easy to illustrate the principle of multichain cross segmentation method by using two chains. As shown in Figure 5, chain1 and chain2 have respectively m and n bins (m≈n), where different rectangles represent different sizes. The bin sizes of the chain are non-uniform, and the two chains are not exactly identical. Moreover, different chains have different physical positions, so the routing delay from the signal input port to each chain is also different. Based on the above two points, the same input signal in different chains pass through different bins. According to Figure 5, the routing delay of the second chain relative to the first chain is $\;\Delta t_{1\to 2}$, and the rising edge of the input signal falls in the *i*-th bin of the first chain and in the *j*-th bin of the second chain. It is equivalent to that of the rising edge of the input signal that falls on the *k*-th bin of the equivalent chain, where *k* = *i* + *j*. We can see that by merging two chains into an equivalent chain, the ultrawide bin are segmented significantly. The averaged bin sizes of chain1 and chain2 are $\tau_{1}=T/m$ and $\tau_{2}=T/n$, respectively. The averaged bin size of the equivalent chain is $\tau_{e}=T/\left(m+n\right)\approx \tau_{1}/2\approx \tau_{2}/2$, which is approximately half the averaged bin size of a single chain.

***Evaluation of the bin size of equivalent chain.*** The actual bin size of the single chain can be obtained through bin-by-bin calibration, and the bin size of the equivalent chain also needs to be evaluated. When calibration hits are fed into both chains, the size of the *i*-th bin in chain1 is $\tau 1_{i}=n_{i}T/N$ and the size of *j*-th bin in chain2 is $\tau 2_{j}=n_{i}T/N.$ Here $n_{i}$ and $n_{j}$ are respectively counts of the hits in *i*-th bin and *j*-th bin while the number of total calibration hits is *N*. The calibration hits which are in both *i*-th bin of chain1 and *j*-th bin of chain2 are regarded as in the *k*-th bin of equivalent chain. Therefore, the size of k-th bin of equivalent chain can be evaluated as

(7) $$\tau_{k}=\frac{n_{k}}{N}T,$$

where $n_{k}$ is the counts of the calibration hits in k-th bin of the equivalent chain. After calibration, the actual bin sizes of the equivalent chain are obtained. The solution with two chains described above can easily be expanded to multiple chains.

***Improvement of uniformity and temperature toleration***. Ideally, when the bins of a chain are uniform, the standard deviation σ is caused by quantization error as

(8) $$\sigma^{2}=\frac{s^{2}}{12}\;,$$

where *s* is the bin size of the uniform chain. Considering that the bin size of actual chain is non-uniform, the σ is written as

(9) $$\sigma^{2}=\sum_{i}(\frac{s_{i}{}^{2}}{12}\times \frac{s_{i}}{S})\;,$$

where $s_{i}$ is the size of the *i*-th bin and $S=\underset{i}{\sum }s_{i}$. The equivalent bin size $s_{ei}$ of equivalent chain can be written as

(10) $$s_{ei}=r_{i}\times s_{i}\;,$$

where $0<r_{i}\leq 1$. The standard deviation of equivalent chain can be written as

(11) $$\sigma_{e}{}^{2}=\sum_{i}(\frac{{\left(r_{i}\times s_{i}\right)}^{2}}{12}\times \frac{r_{i}\times s_{i}}{S})\;.$$

From (9), we can see that the contribution of a bigger bin is larger than smaller bin. After segmentation, the $r_{i}$ of bigger bin is smaller. It can be seen from (11) that the stability of the equivalent chain is improved. The deterioration in stability brought about by the bigger bin is particularly weakened.

From the introduction in Section 2.2, We can consider that the temperature fluctuation has the same effect on each bin, assuming the temperature coefficient is k. The bin size affected by temperature of plain chain can be written as

(12) $$s_{ti}=k\times s_{i}\;.$$

The equivalent bin size $s_{tei}$ of the equivalent chain can be written as

(13) $$s_{ti}=k\times r_{i}\times s_{i}\;.$$

From (13), we can see that the variation of the equivalent bin size is smaller than plain bin size with the temperature fluctuation. It can also be said that the variation of equivalent resolution caused by temperature fluctuation is smaller. Finally, it is reflected on the TDC with a low temperature coefficient.

## 3. Results

### 3.1. Implementation of the Multichain TDC

**Figure 6:**
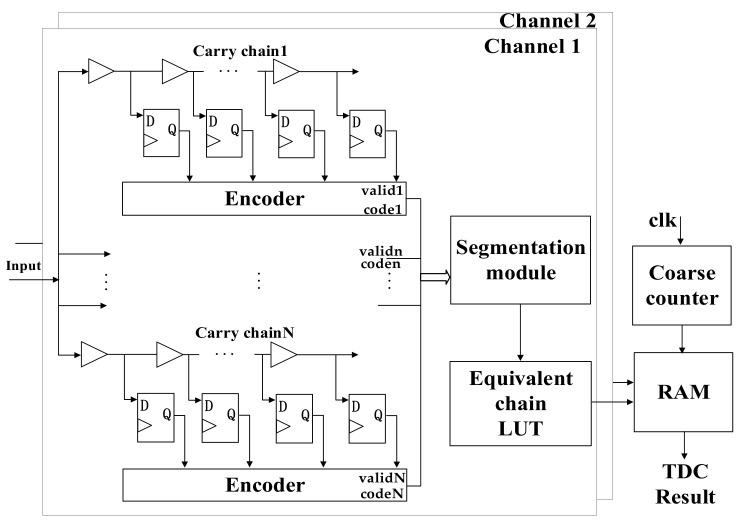
Block diagram of 2-channel TDC based on the multichain cross segmentation.

**Figure 7:**
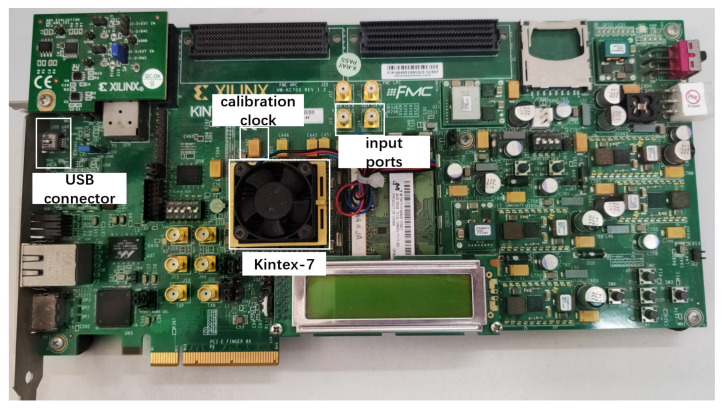
A picture of the evaluation board.

A 2-channel TDC based on a multichain cross segmentation method is implemented in XC7K325T device from the Xilinx Kintex-7 family. The architecture of the TDC is shown in Figure 6. Each channel is composed of multiple parallel carry chains for fine time measurement. When the encoding is completed, the binary code and valid signal from each chain will be sent to segmentation module, which converts them to binary code of the equivalent chain. A counter is used to record the number of clock periods for coarse time measurement. The clock frequency is 250 MHz. The time interval between the two channel signals can be calculated by coarse time and fine time, which are stored in the RAM. Figure 7 shows the photograph of our evaluation board. The calibration signals for bin-by-bin calibration are generated by the internal ring oscillator with a frequency of 156.25 MHz and this calibration clock has no correlation with the system clock with a frequency of 200 MHz. The USB connector sends the results to a computer. Two SMA connectors are used to receive the hit signals.

### 3.2. Averaged Resolution and Nonlinearity

**Figure 8:**
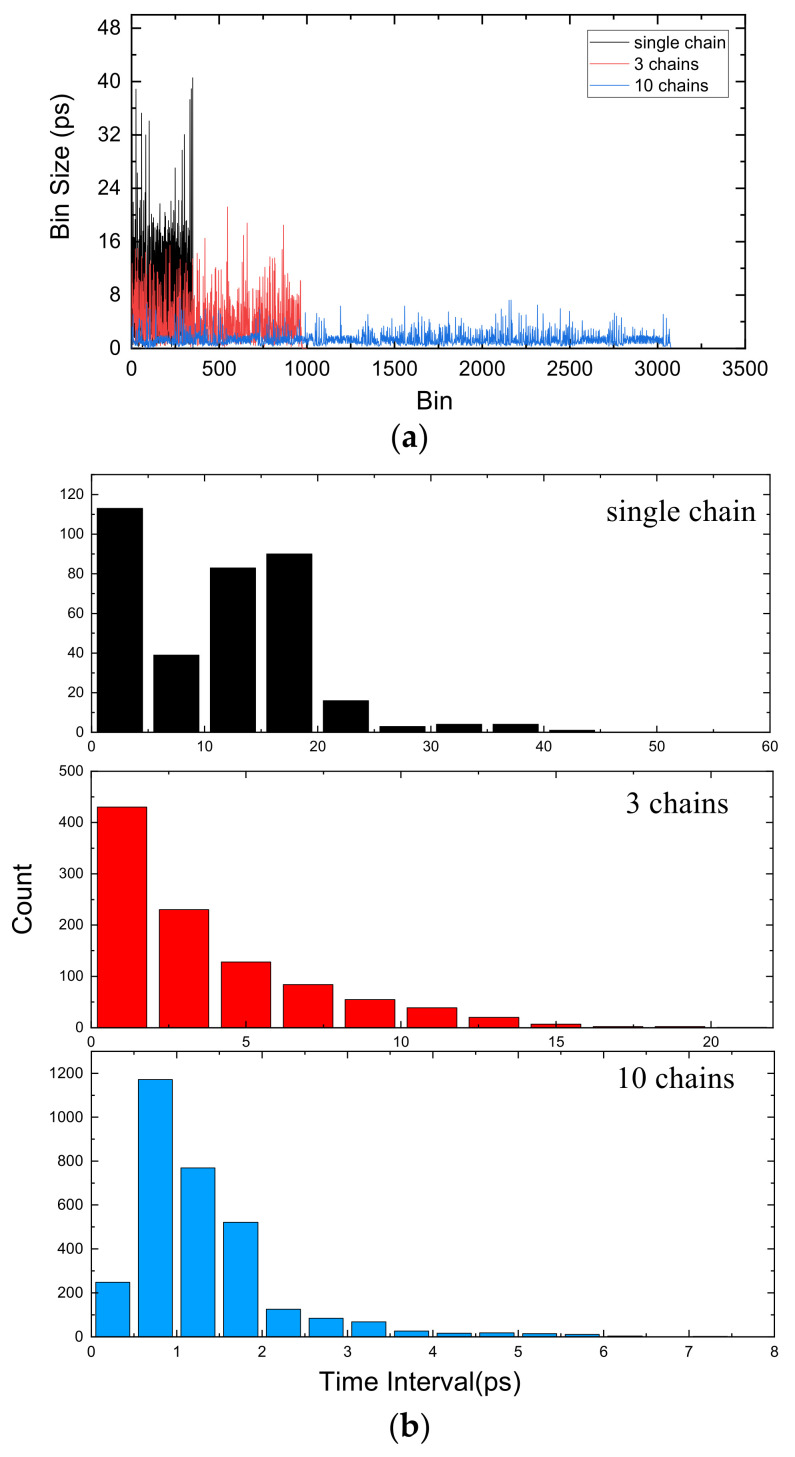
(**a**) Bin size with different chain numbers. (**b**) Histogram of bin size.

We applied a different number of chains to test the improvement of multichain cross segmentation. The TDC bin size is obtained by bin-by-bin calibration. We used an oscillator independent of the system clock to generate $10^{8}$ test hits. The bin size with different chain numbers is shown in Figure 8a and the histogram of bin size is shown in Figure 8b, from which we can see that the bin size is effectively subdivided as the number of chains increases. The 4 ns period contains 350 interpolation units in single chain, so that the averaged resolution can be calculated as 1 LSB = 4000/350 = 11.4 ps. The averaged resolution is reduced to 4.1 ps with 3 chains and is further reduced to 1.3 ps with 10 chains. However, the average resolution cannot be reduced indefinitely. As the number of chains increases, the gain gradually decreases.

**Figure 9:**
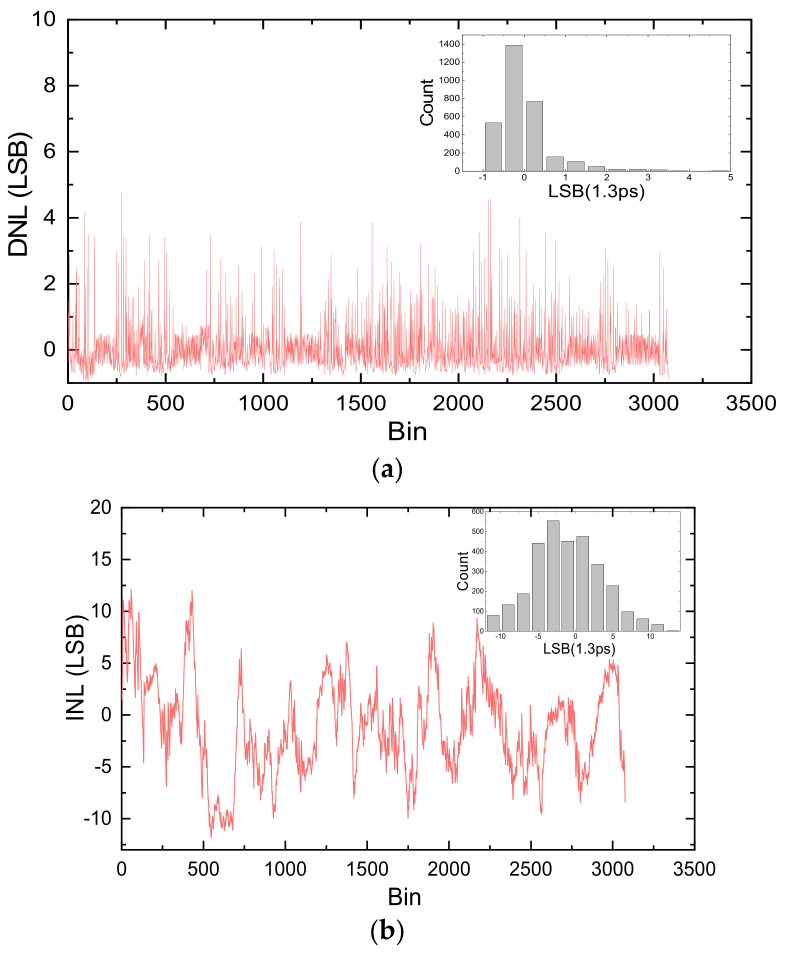
Nonlinearity of the TDC with 10 chains, (**a**) differential nonlinearity, (**b**) integral nonlinearity.

Differential nonlinearity (DNL) and integral nonlinearity (INL) are used to characterize the nonlinearity of TDC. DNL is defined as the difference between the actual bin size and the averaged bin size, and INL is the sum of DNL from the first bin to the current bin, which can be calculated according to the bin size information. The DNL and the INL of 10 chains are shown in Figure 9a,b, respectively. The DNL is from the range of (−0.99, 4.79) LSB, and the INL is from the range of (−14.18,13.16) LSB.

### 3.3. RMS Precision

**Figure 10:**
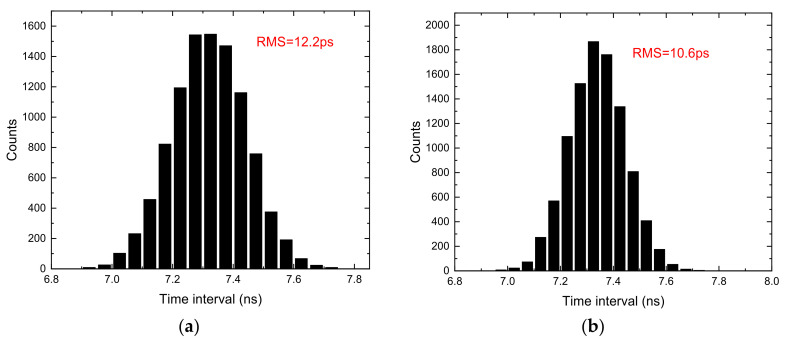

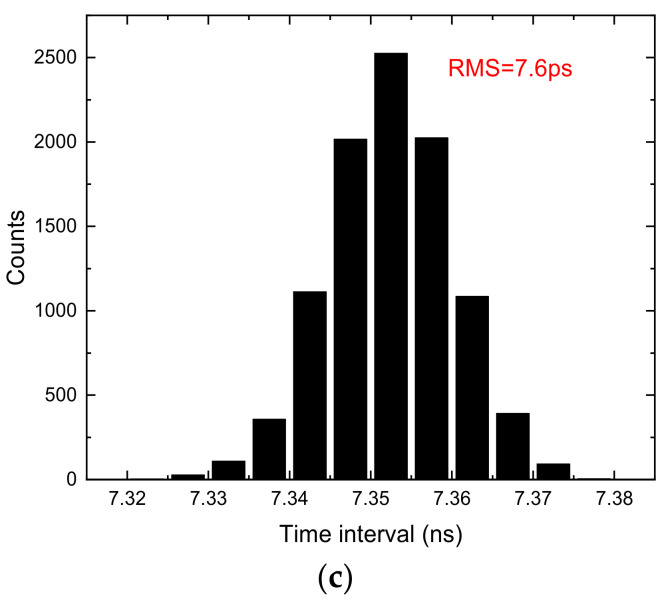
RMS precision of the TDC with different chain number. (**a**) Chain number = 1; (**b**) chain number = 3; (**c**) chain number = 10.

In addition to the averaged resolution and nonlinearity, TDC has a more important index: RMS precision. The standard deviation of the measurement results for a fixed time interval is used to characterize the RMS precision. The cable delay method is usually used to measure the RMS precision [18]. The hits for the two channels are fed by the signal generator through T-SMA connectors and cables. Figure 10 shows the RMS precision of the time interval measurement with different chains. In Figure 10a the RMS is 12.2 ps with single chain. From Figure 10b,c we can see that the RMS is improved up to 10.6 ps and 7.6 ps with 3 chains and 10 chains, respectively. The RMS value is improved with the increase of the number of chains.

**Figure 11:**
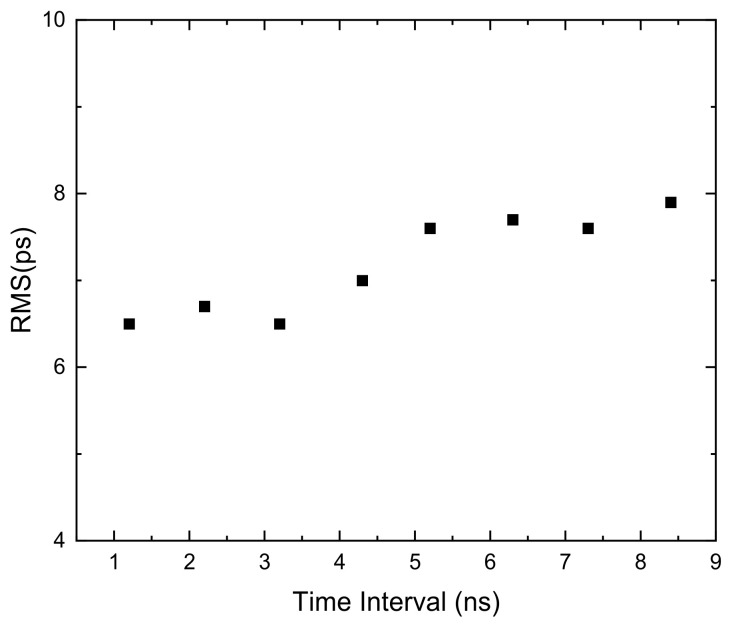
RMS precision of the 10-chain TDC with different time interval.

The RMS for different time intervals is measured with 10 chains, as shown in Figure 11. The precision of large-scale time intervals depends on fine measurement. Therefore, we only need to measure the time intervals covering one clock period (4 ns). The measurement results show that for the time intervals from 1 ns to 9 ns, the RMS is roughly the same, and reaches 6.5 ps at its peak. The single-shot precision of the single channel is 4.6 ps which equals $\mathrm{RMS}/\sqrt{2}$.

**Figure 12:**
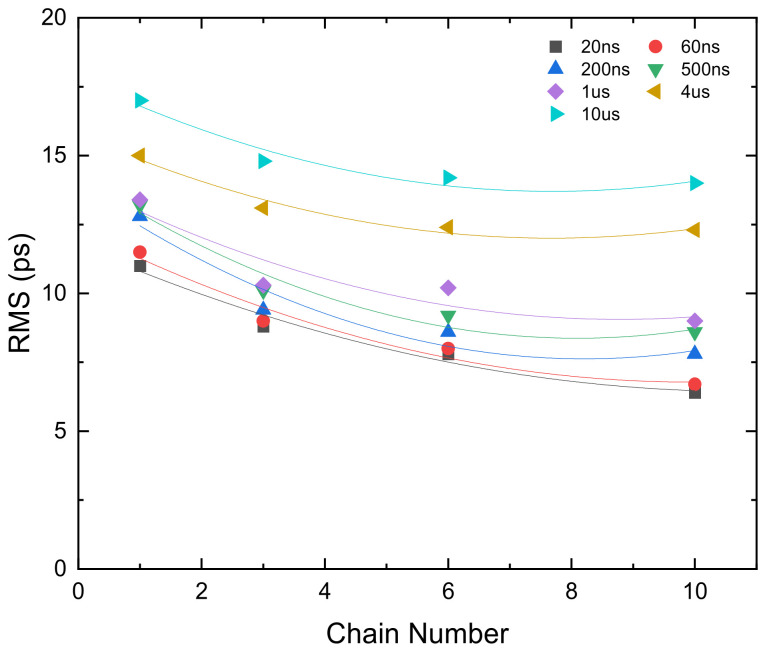
RMS precision of the TDC with different chain numbers for different time intervals.

To observe the improvement brought by the multichain segmentation, we also used TDCs with different number of chains to measure different time intervals. The results of RMS precision for these TDCs are compared in Figure 12. The RMS precision of the short time interval increases as the number of chains increases. For example, the RMS values of 60 ns time interval with single chain, 6-chain, 10-chain are 11.5 ps, 8 ps, and 6.7 ps, respectively. With the increase of time interval, the RMS precision deteriorates, and the extent of improvement shows saturation characteristics as the number of chains increases. The RMS values of 4 us time interval with single chain, 6-chain, 10-chain are 15 ps, 12.4 ps, and 12.3 ps, respectively.

In addition to the clock jitter between the two channels, the clock error caused by the time interval and clock stability will also degrade the RMS value. The coarse time can be expressed as

(14) $$T_{coarse}=\left(N-M\right)T_{clk},$$

where the number of the cycle $T_{clk}$ included in the coarse time is  N−M. Considering clock error, from (14) we can get

(15) $$\Delta T_{coarse}=\Delta \left(N-M\right)T_{clk}+\left(N-M\right)\Delta T_{clk}$$

Then

(16) $$\frac{\Delta T_{coarse}}{T_{coarse}}=\frac{\Delta \left(N-M\right)}{\left(N-M\right)}+\frac{\Delta T_{clk}}{T_{clk}}.$$

According to the principle of electronic counting, $\Delta \left(N-M\right)$ = ±1, $N-M=T_{coarse}/T_{clk}$, from (16) we can get

(17) $$\Delta T_{coarse}=\pm T_{clk}+T_{coarse}\frac{\Delta T_{clk}}{T_{clk}}.$$

where $T_{coarse}\cdot \Delta T_{clk}/T_{clk}$ is clock error. The clock error caused by the clock stability will become worse when the time interval becomes longer, resulting in measurement precision degradation.

### 3.4. Temperature Variation

**Figure 13:**
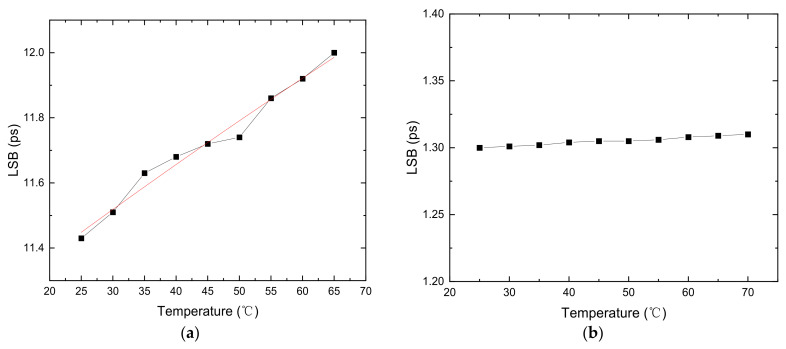
LSB of the TDC with different chain number varying with ambient temperature. (**a**) Chain number = 1; (**b**) chain number = 10.

As mentioned above, the multichain cross segmentation TDC will reduce the effect of temperature variation. We observed bin size variations of plain TDC and multichain segmentation TDC with 10 chains in the operating temperature range of 25 °C to 70 °C. The FPGA chip was heated with a hot air gun. The operating temperature was measured with an XADC with an integrated sensor and can monitor the temperature of the FPGA chip in real time. At each test temperature point, we maintained the temperature for a period of time and measured it many times during this period. As shown in Figure 13a, we observed that the bin size of plain TDC varied almost linearly versus temperature. For the temperature from 25 °C to 70 °C, the LSB increases from 11.43 ps to 12.05 ps. The temperature coefficient is 0.014 ps/°C. Bin size of 10 chains TDC versus temperature is shown as Figure 13b. The LSB is almost the same with different temperature. The LSB increases from 1.30 ps to 1.31 ps with the temperature from 25 °C to 70 °C. The temperature coefficient reduced to 0.0002 ps/°C. The results show that the TDC using the multichain cross segmentation method has a very low temperature coefficient, which effectively improves the effect of temperature variation.

### 3.5. Resource Usage and Power Consumption

The resource occupation and power consumption of the TDC with one-chain and ten-chain are shown in Table 1. The resources used primarily included LUT, flip-flop registers, random-access-memory block (BRAM) and phase-locked loops (PLLS). The 10-chain TDC occupy more resources compared with plain TDC mainly for more chains.

## 4. Discussion

### 4.1. Features of the Multichain Cross Segmentation Method

**Figure 14:**
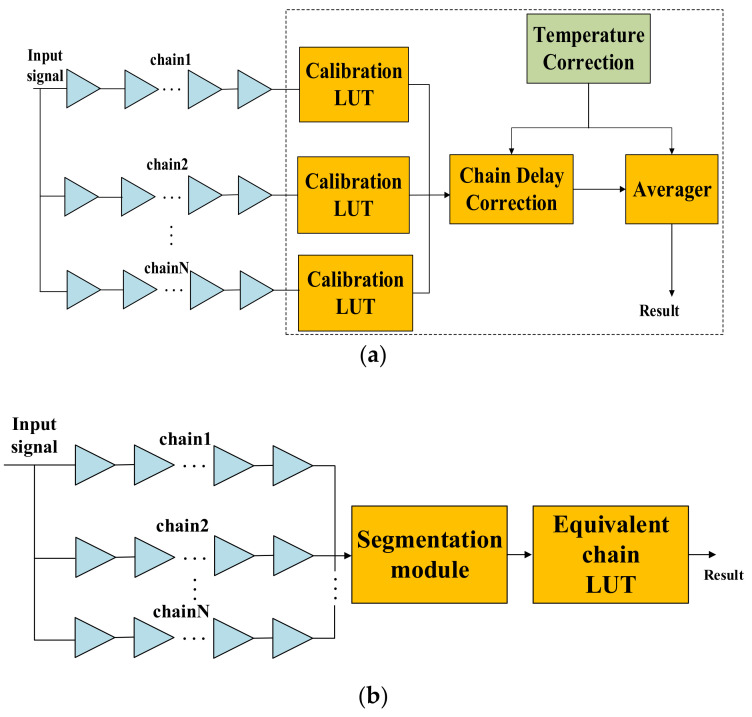
Block diagram (**a**) multichain measurements averaging; (**b**) multichain cross segmentation.

The multichain cross segmentation method is fundamentally different from the multichain measurements averaging method used in ref. [20]. It is mainly reflected in three aspects: resolution, architecture, and temperature coefficient. The block diagram of multichain measurements averaging is shown in Figure 14a. Multiple parallel chains are used to measure the input signal simultaneously, and the average of multiple measurements is taken as the final fine time. Theoretically, the resolution is improved by $\sqrt{N}$ times, where N is the number of parallel chains. The block diagram of multichain cross segmentation is shown in Figure 14b. N chains are merged into an equivalent chain through mutual segmentation, which is equivalent to increasing the number of bins for interpolating one clock period, and the resolution is improved by N times. Secondly, the multi-chain measurements averaging method requires us to build calibration LUTs for each chain, and the delay between different chains also needs to be calibrated before averaging. On the contrary, the multichain cross segmentation method only needs one LUT for the equivalent chain, and the timestamp can be obtained directly without multitime measurements. Thirdly, the bin size of plain chain is affected by temperature variation, which cannot be reduced by averaging, so this architecture needs a corresponding calibration strategy. However, the bin size variation of plain chain due to temperature can also be segmented by other chains. After multichain cross segmentation, we can obtain an equivalent chain with fine granularity and uniformity bins. Therefore, the effect of temperature variation will be reduced significantly.

The TDC implemented by multichain cross segmentation method uses ordinary logic cells, and there will be no large difference in ordinary logic cells between different FPGA devices. Therefore, the multichain cross segmentation method presented in this paper is probably applicable to other devices, although it was only validated in Kintex-7 FPGA in this paper.

### 4.2. Resolution and Precision

Resolution and precision are two important indicators to measure the performance of TDC. Resolution, also known as the least significant bit (LSB), is the minimum time that can be measured [26]. Precision, standard deviation of the measurement results for a fixed time interval, is used to characterize the fluctuation of measurements. After multichain cross segmentation, the ultrawide bins are effectively segmented, however, the nonlinearity still exists. Thus, the precision is not improved to the same extent as the averaged resolution. Bin-by-bin calibration can obtain the actual width of each bin so it can eliminate the measurement error caused by nonlinearity. In fact, when the number of TDLs is more than six, the precision has no significant improvement anymore. If the nonlinearity can be improved, the precision will be further improved.

The carry chain has ultrawide bins because of clock skew caused by clock tree at the regional boundary. Raising clock frequency will shorten the length of the carry chain. This can avoid the chain crossing the clock regions, thereby improving nonlinearity, and reducing the resource occupation. The encoder determines the clock frequency of the TDC circuit. In our work, considering the performance, the encoder implemented uses a three-stage pipeline structure. The maximum clock frequency of the TDC is 250 MHz, and a 4 ns period corresponds to approximately 350 bins so that the chain would cross the clock region in a Kintex-7 device. Optimizing the encoder or using a device with more resources, such as a Virtex-7 FPGA, can avoid crossing clock regions and improve the precision.

### 4.3. Accuracy

**Figure 15:**
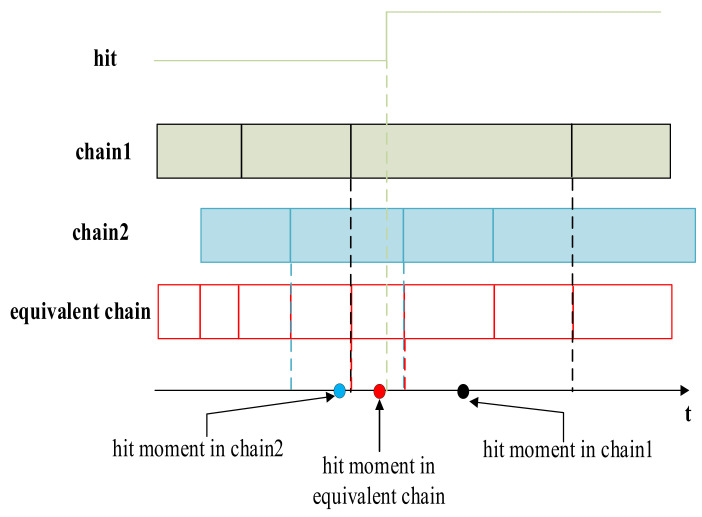
Point to the hit moment on time axis of different chain.

The accuracy can be evaluated from two aspects: an experimental test of standard devices and theoretical analysis. Here, a brief accuracy evaluation is carried out through the analysis of quantization error, and the detailed evaluation will be carried out through experimental tests in practical application. The calibration method explained in Section 2 allowed accurate evaluation of the bin size of carry chain according to Equation (2). The accuracy of measurement is caused by quantization error. The last bin contributes half the size in the time interval calculation according to Equation (5). The point on the time axis corresponding to the moment of the hit signal is shown in Figure 15. The maximum deviation of the hit moment is half of the current bin size. As per the experiments above, the maximum bin sizes of single chain and 10-chain are 56 ps and 7.53 ps, respectively. Therefore, the accuracies of the single chain TDC and 10-chain TDC are 28 ps and 3.77 ps, respectively.

## 5. Conclusions

We have proposed a multichain cross segmentation method for carry chain-based TDC. By merging multiple parallel carry chains into an equivalent chain, the resolution and precision can both be improved and the performance made insensitive to operating temperature. A 2-channel multichain cross segmentation TDC is implemented with 10 chains in Kintex-7 FPGA with 1.3 ps resolution and 4.6 ps single-shot precision, which can achieve a temperature coefficient of 0.0002 ps/°C in a temperature range from 25 °C to 70 °C. Each channel has a 125 M samples/second (MS/s) measurement rate. Table 2 provides a comparison with other FPGA-TDC. Our work has achieved a high performance, including LSB, precision, dead time, temperature tolerance and so on. This TDC can be used in systems such as optical fiber time–frequency transmission, to realize accurate measurement of time interval and improve system integration.

## Figures and Tables

**Table 1:** Resources usage and power consumption of the TDC.

Resource	Available	1-Chain		10-Chain	
		Use	Use %	Use	Use %
LUT	203,400	826	0.4	6299	3.1
FF	406,800	1727	0.42	14,334	3.5
BRAM	445	1	0.22	3	0.67
Power		0.482 w		0.563 w	

**Table 2:** FPGA-TDC performance comparison.

Ref.	Method	Device	LSB [ps]	Precision [ps]	DNL [LSB]	INL [LSB]	Dead Time [ns]	Temperature Correction	Resources	Power [w]
[31]	Vernier-type	Kintex-7	43	34	−0.98, +0.08	−1, +0.97	50	NA	NA	NA
[27]	Multi-chain averaging	Kintex-7	11	15	−1, +1.4	−1.75, +3.5	NA	NA	NA	NA
[32]	NUMMP ^(1)^	Kintex-7	1.87	2.79	−0.54, +1.3	−2.21, +3.51	8	Re-TSM ^(6)^	1103FFs+ 1679LUTs	0.740
[33]	LSPM ^(2)^	Kintex-7	1.29	3.54	−1.2, +1.4	−3.28, +3.78	NA	NA	3900FFs+ 1002LUTs	0.453
[28]	Wave union +Bin Realignment	Kintex-7	NA	8.7	0, +4.6	NA	1.47	Calibration table updating	NA	NA
[29]	Multi-measurement	Kintex-7	3	5.76	−1, +3.3	−8, +9	22	Calibration table updating	NA	NA
[34]	Multi-phase	Kintex-7	78.13	35	−0.28, +0.53	−0.56, +0.38	<50	NA	347FFs+ 199LUTs	NA
[26]	NUMP ^(3)^	Cyclone-V	2.3	8.8	−0.81, +1.20	NA	NA	LUT auto-updating	11433LCs+ 7127FFs	NA
[22]	Multi-chain averaging	Virtex-7	1.15	3.5	−0.98, +3.5	−5.9, +3.1	8	Curve fitting	19666LUTs	NA
[25]	Wave union	Cyclone II	22	30	−0.99, +2.18	NA	NA	Dedicated correction channel	23494LCs+ 28085FFs	NA
[24]	TDL ^(4)^	Virtex-4	~50	25	−0.4, +0.6	−1.3, +1.7	10	Coefficient compensation	NA	NA
This work	MCS ^(5)^	Kintex-7	1.3	4.6	−0.99, +4.79	−14.18, +13.16	8	Self-adaption	14334FFs+ 6299 LUTs	0.563

^(1)^ NUMMP = nonuniform monotonic multiphase; ^(2)^ LSPM = large-scale multiphase matrix; ^(3)^ NUMP = non-uniform multiphase; ^(4)^ TDL = tapped delay line; ^(5)^ MCS= multichain cross segmentation; ^(6)^ Re-TSM = remarking the time scales method.

## Data Availability

Not applicable.
